# Cyberbullying among college students in a Chinese population: Prevalence and associated clinical correlates

**DOI:** 10.3389/fpubh.2023.1100069

**Published:** 2023-02-22

**Authors:** Xingyue Jin, Kun Zhang, Mireille Twayigira, Xueping Gao, Huiming Xu, Chunxiang Huang, Xuerong Luo, Yanmei Shen

**Affiliations:** ^1^Department of Psychiatry, National Clinical Research Center for Mental Disorders, The Second Xiangya Hospital of Central South University, Changsha, Hunan, China; ^2^College of Physical Education, Changsha University of Science and Technology, Changsha, Hunan, China

**Keywords:** cyberbullying, online bullying, bullying, Chinese college students, risk factors, prevalence

## Abstract

**Objectives:**

Cyberbullying is quite common in adolescents and college students, and it influences mental health in many aspects. The purpose of this study was to investigate the prevalence of cyberbullying in Chinese college students and to look for related factors.

**Methods:**

Eight thousand and ninety-eight college students aged 17–26 were enrolled in this cross-sectional study. We collected information of their sociodemographic data, depression (evaluated by Self-Rating Depression Scale), anxiety (evaluated by Self-Rating Anxiety Scale), lifetime suicidal behaviors (including suicidal ideation, suicidal plans, and suicide attempts), and experiences of cyberbullying for the past 12 months by online questionnaires.

**Results:**

The prevalence of cyberbullying for the past 12 months was 7.82% (633/8,098) among college students. Binary logistic regression analysis showed that sex (odds ratio, OR = 0.522, 95%CI = 0.433–0.629, *p* < 0.001), suicide attempts (OR = 2.164, 95%CI = 1.589–2.948, *p* < 0.001), depression (OR = 2.372, 95%CI = 1.602–3.512, *p* < 0.001), and anxiety (OR = 1.911, 95%CI = 1.305–2.800, *p* = 0.001) were independently associated with cyberbullying.

**Conclusion:**

Cyberbullying is very common among college students in Hunan Province, China. Besides, being male, suicide attempts, depression and anxiety were independently associated with cyberbullying, which highlights the importance of paying attention to cyberbullying and addressing anxiety, depression, and suicidal behaviors among college students to better improve their mental health and prevent suicide.

## 1. Introduction

Cyberbullying, sometimes called online bullying or electronic bullying, is bullying with digital approaches, like mobile phones, computers, and tablets (1). Cyberbullying can take place on social media, messaging, gaming (2) or other platforms where people can view, participate in, or share content (1). Among these, the most common place where cyberbullying takes place is on social media, like Facebook, Instagram, Snapchat, Tik Tok (1), and Weibo in China (3). Huston et al. summarized that the defining characteristics of cyberbullying are: (a) “electronic form of contact” (b) “an aggressive act” (c) “intent” (d) “repetition (publicity),” and (e) “harm to the victim” (4). There are different forms of cyberbullying, such as flaming, online harassment, cyberstalking, denigration, impersonation, disclosure of private information (outing), and exclusion (5). For example, cyberbullying could involve sharing and spreading personal or private information which leads to embarrassment or humiliation (1), sending negative, hurtful, false, mean messages or threats on messaging platforms (1, 2). Significantly, face-to-face bullying and cyberbullying can sometimes happen simultaneously (2), and some cyberbullying behaviors are so egregious that they turn into unlawful or criminal behavior (1). Compared with bullying in the traditional sense, cyberbullying has some unique characteristics: (a) persistent, the internet makes it possible to immediately and continuously spread 24 h a day; (b) permanent, most information on the internet is permanent and public if not removed; (c) hard to recognize for teachers and caregivers when it takes place (1). Cyberbullying could have a negative impact on an individual mentally, physically, and emotionally. People who have suffered cyberbullying may feel upset, embarrassed, ashamed, angry, or lose interest in the things they love, and they may sometimes have sleep problems or somatic symptoms like headaches or stomachaches, and in extreme cases, cyberbullying can even lead to people taking their own lives (2).

Previous studies have revealed that cyberbullying is quite common among youth. In 2017, about 15% of American students in grades 9–12 reported having been cyberbullied in the past 12 months (6). In 2019, the Cyberbullying Research Center found that for middle and high school students aged 12–17 in the United States, approximately 37% had experienced cyberbullying in their lifetime (7), indicating that cyberbullying is quite common and shows a growing trend. Relative to numerous studies among adolescents, less evidence was found among youth at early adulthood. A review showed that about 10–15% students had suffered cyberbullying during their college (8). Alrajeh et al. reported 35.8% students in Qatar University were cyberbully-victims (9). Huang et al. found a high prevalence of cyberbullying up to 64.32% in Chinese college students (10). In addition, cyberbullying has been reported to have many adverse effects on one's mental health. For example, Grigore et al. found significant positive associations between cyberbullying and anxiety in middle school students (11). Meanwhile in other studies, cyberbullying victims presented with high levels of anxiety, stress (12), and social anxiety (13). In prior studies, people who had been cyberbullied also had significantly higher levels of depression (14–16). In a study done in Bangladesh, the prevalence of major depressive disorder in cyberbullying victims was 9.1%, proportionately higher than that in non-victims (17). Cole et al. found that cybervictimization significantly predicted depressive symptoms (15), and multivariate analysis showed that college students who had experienced cyberbullying had a higher likelihood of being depressed and anxious than those without cyberbullying experience. Additionally, Jin et al. found that Chinese middle school students in Chongqing city who were being cyberbullied were more likely to have suicidal attempts (18).

Cyberbullying not only influences current mental health, but may also affect mental health in the long term. However, the direction of the causal relationship between cyberbullying and psychological problems is still less clear. Lee found that childhood cyberbullying experiences increased the likelihood of subsequent cyberbullying victimization and cyberbullying perpetration and escalated the risk of anxiety (19), while Liu et al. found that emotional distress at baseline was associated with cyberbullying after 8 months (20).

Previous studies on youth cyberbullying have mainly been conducted among teenagers, and in college students from several countries. It should be noted that, as has mentioned above, college students are at a high risk of suffering from cyberbullying. As the prevalence and correlates of cyberbullying are greatly influenced by national and cultural factors (21), it is necessary to elucidate the study of cyberbullying in different regions and countries, especially in a large sample size. Few studies have investigated cyberbullying in a large sample size of Chinese college students. Compared with other young populations in China, college students have more access to the internet, chat online more, and they browse and share perspectives with friends or strangers online more than other groups, which makes them more susceptible to cyberbullying. Therefore, the purpose of this study was to investigate the prevalence of cyberbullying in a large sample of Chinese college students and to look for related factors.

## 2. Methods

### 2.1. Participants

This study was ethically approved by the Ethics Committee of Second Xiangya Hospital of Central South University. A cross-sectional design was employed in this study. We designed a questionnaire and distributed it online. From February to June 2019, data were collected from college students at Changsha Medical University and Changsha University of Science and Technology. Students who met the following inclusion criteria were invited to join this study: (a) aged 17–26; (b) not suffering from any severe physical illness; (c) willing to sign a formal consent form to participate. Before the survey, students were introduced to the purposes, contents, and form of the study, and were told that they had the right to participate or not, or to quit at any time, and that their information would be kept and treated with strict confidence. Using convenience sampling, a total of 8,130 students were invited to join he study, 6,045 students from Changsha University of Science and Technology, and 2,085 students from Changsha Medical University. Among the students, 32 refused to participate or did not complete the whole survey. Therefore, 8,098 students participated in the study and were included in the subsequent analyses. The response rate was 99.6%.

### 2.2. Measurements

In the questionnaire, we surveyed participants' sociodemographic data, mental states, and conditions about cyberbullying. The sociodemographic data included their age, sex, community, nationality, physical disorder history, mental disorder history, family history of mental disorders (FHMD), right-handedness, good relationship with mother, good relationship with father, single-child household (yes or no), family income, smoking, and alcohol drinking.

The symptoms of anxiety in the past 7 days were measured by the 20-item Self-Rating Anxiety Scale (SAS), and SAS scores higher than 50 were regarded as having anxiety (22). The symptoms of depression in the past 7 days were measured by the Self-Rating Depression Scale (SDS), and SDS scores higher than 53 were regarded as having depression (23). These two scales have shown good reliability and validity in Chinese population (24, 25).

Suicidal behaviors were measured by the following questions: (a) suicidal ideation: “Have you ever seriously thought about committing suicide?” (b) suicidal plans: “Have you ever made a plan about how you would commit suicide?” (c) suicidal attempts: “Have you ever tried to commit suicide?” For each question, participants were asked to choose “yes” or “no”.

We firstly defined cyberbullying to participants: “Bullying is the behavior where an individual is being repeatedly and persistently treated negatively by one or more people who are intentionally causing or attempting to cause harm and discomfort to the individual. When bullying happens, the bullied individual is clearly at a disadvantage. Cyberbullying is the form of bullying using electronic means.” Cyberbullying was then measured by the following question: “In the past 12 months, has someone used the internet, SMS, Weibo, WeChat (or other electronic devices) to bully, tease or threaten you?” Participants were asked to choose the frequency of cyberbullying: (a) never; (b) once; (c) twice; (d) three times or more. Students who experienced cyberbullying at least once were regarded as cyberbullying victims.

### 2.3. Statistical analysis

In this study, *t-*test was used to compare the group differences between students with and without cyberbullying for normally distributed continuous data such as age, SAS scores, SAS scores. Chi-square test was used to compare group differences for categorical data such as sex, nationality. Binary logistic regression analysis was conducted for adjusted odds ratio (OR) calculation for cyberbullying. In addition, spearman's correlation analysis was utilized for the correlations between cyberbullying scores, sociodemographic data, and mental states, and Bonferroni corrections was used for multiple comparisons testing. Stepwise multiple regression was used to further examine the association between cyberbullying score and other variables. All statistical analyses were conducted in SPSS (Version 22.0; IBM, Inc., Chicago, IL). The significance level was set at 0.05 (two-tailed).

## 3. Results

The prevalence of cyberbullying was 7.82% (633/8,098) in the present study. No significant differences were found between cyberbullying victims and non-victims in the following demographic characteristics: age, community, nationality, right-handedness, and only-child status (all *p* > 0.05, Table 1). However, cyberbullying victims were more likely to have FHMD or have a habit of smoking (both *p* = 0.001). In addition, participants who suffered cyberbullying were more likely to be male, have physical or mental disorder history, have poorer relationship with parents, drink alcohol frequently, be depressed or anxious (consistent with their significantly higher scores on SDS and SAS), and they were more likely to have suicidal behaviors, including suicidal ideation, suicide plans, and suicide attempts (all *p* < 0.001, Table 1). More of the victims of cyberbullying also had a family annual income lower than 30,000 compared with non-victims (*p* = 0.005).

**Table 1:** Comparison of demographics and clinical characteristics between cyberbullying victims and non-victims.

**Variable**	**Non-victim (*n* = 7,465)**	**Victim (*n* = 633)**	***P*-value**	**OR (95% CI)**
Age (years), mean ± SD	20.23 ± 1.50	20.34 ± 1.48	0.069	
Sex				
Males, *n* (%)	3,220 (43.1%)	372 (58.8%)	0.000	0.532 (0.451–0.628)
Females, *n* (%)	4,245 (56.9%)	261 (41.2%)		
Community				
Urban	3,193 (42.8%)	249 (39.3%)	0.093	
Rural	4,272 (57.2%)	384 (60.7%)		
Nationality				
Han	6,777 (90.8%)	581 (91.8%)	0.401	
Others	688 (9.2%)	52 (8.2%)		
Physical disorder history, *n* (%)	222 (3.0%)	37 (5.8%)	0.000	2.025 (1.416–2.897)
Mental disorder history, *n* (%)	92 (1.2%)	20 (3.2%)	0.000	2.615 (1.601–4.270)
FHMD, *n* (%)	90 (1.2%)	18 (2.8%)	0.001	2.398 (1.436–4.005)
Right-handedness, *n* (%)	6,712 (89.9%)	561 (88.6%)	0.304	
Good relationship with mother, *n* (%)	7,214 (96.6%)	587 (92.7%)	0.000	0.444 (0.321–0.615)
Good relationship with father, *n* (%)	7,094 (95.0%)	576 (91.0%)	0.000	0.528 (0.395–0.707)
Only-child, *n* (%)	2,982 (39.9%)	258 (40.8%)	0.689	
SDS	43.94 ± 9.70	51.48 ± 10.86	0.000	
Depression, *n* (%)	772 (10.3%)	245 (38.7%)	0.000	5.474 (4.589–6.531)
SAS	39.40 ± 8.92	47.79 ± 11.94	0.000	
Anxiety, *n* (%)	1,051 (14.1%)	279 (44.1%)	0.000	4.810 (4.058–5.701)
Family income/year (yuan)				
< 30,000, *n* (%)	1,848 (24.8%)	184 (29.1%)	0.005	
30,000~70,000, *n* (%)	3,045 (40.8%)	268 (42.3%)		
More than 70,000, *n* (%)	2,572 (34.5%)	181 (28.6%)		
Smoking, *n* (%)	651 (8.7%)	80 (12.6%)	0.001	1.514 (1.182–1.940)
Alcohol drinking, *n* (%)	2,311 (31.0%)	272 (43.0%)	0.000	1.680 (1.425–1.982)
Suicidal ideation, *n* (%)	1,523 (20.4%)	227 (35.9%)	0.000	2.181 (1.837–2.591)
Suicide plans, n (%)	245 (3.3%)	71 (11.2%)	0.000	3.723 (2.820–4.915)
Suicide attempts, n (%)	428 (5.7%)	124 (19.6%)	0.000	4.005 (3.217–4.987)

FHMD, family history of mental disorders; SAS, Self-Rating Anxiety Scale; SDS, Self-Rating Depression Scale.

In addition, correlation analysis showed significant correlations between cyberbullying score and the following parameters: sex (*r* = −0.086, df = 8,098, *p* < 0.001), physical disorder history (*r* = 0.045, df = 8,098, *p* < 0.001), mental disorder history (*r* = 0.046, df = 8,098, *p* < 0.001), FHMD (*r* = 0.040, df = 8,098, *p* < 0.001), good relationship with mother (*r* = −0.057, df = 8,098, *p* < 0.001), good relationship with father (*r* = −0.049, df = 8,098, *p* < 0.001), suicidal ideation (*r* = 0.101, df = 8,098, *p* < 0.001), suicide plans (*r* = 0.113, df = 8,098, *p* < 0.001), suicide attempts (*r* = 0.150, df = 8,098, *p* < 0.001), depression symptoms (*r* = 0.188, df = 8,098, *p* < 0.001), anxiety symptoms (*r* = 0.201, df = 8,098, *p* < 0.001), family income (*r* = −0.036, df = 8,098, *p* = 0.001), smoking (*r* = 0.038, df = 8,098, *p* = 0.001) and drinking alcohol (*r* = 0.071, df = 8,098, *p* < 0.001). All these significant associations passed the Bonferroni corrections (all *p* < 0.05/19 = 0.003). Further multiple regression showed significant associations between the cyberbullying score and anxiety symptoms (beta = 0.179, *t* = 10.499, *p* < 0.001), suicide attempts (beta = 0.085, *t* = 6.856, *p* < 0.001), sex (beta = −0.094, *t* = −8.814, *p* < 0.001), suicide plans (beta=0.046, t=3.733, *p* < 0.001), FHMD (beta=0.031, t=2.868, *p*=0.004), SDS (beta = 0.037, *t* = 2.149, *p* = 0.032) and family income (beta = −0.022, *t* = −2.072, *p* = 0.038).

**Figure 1:**
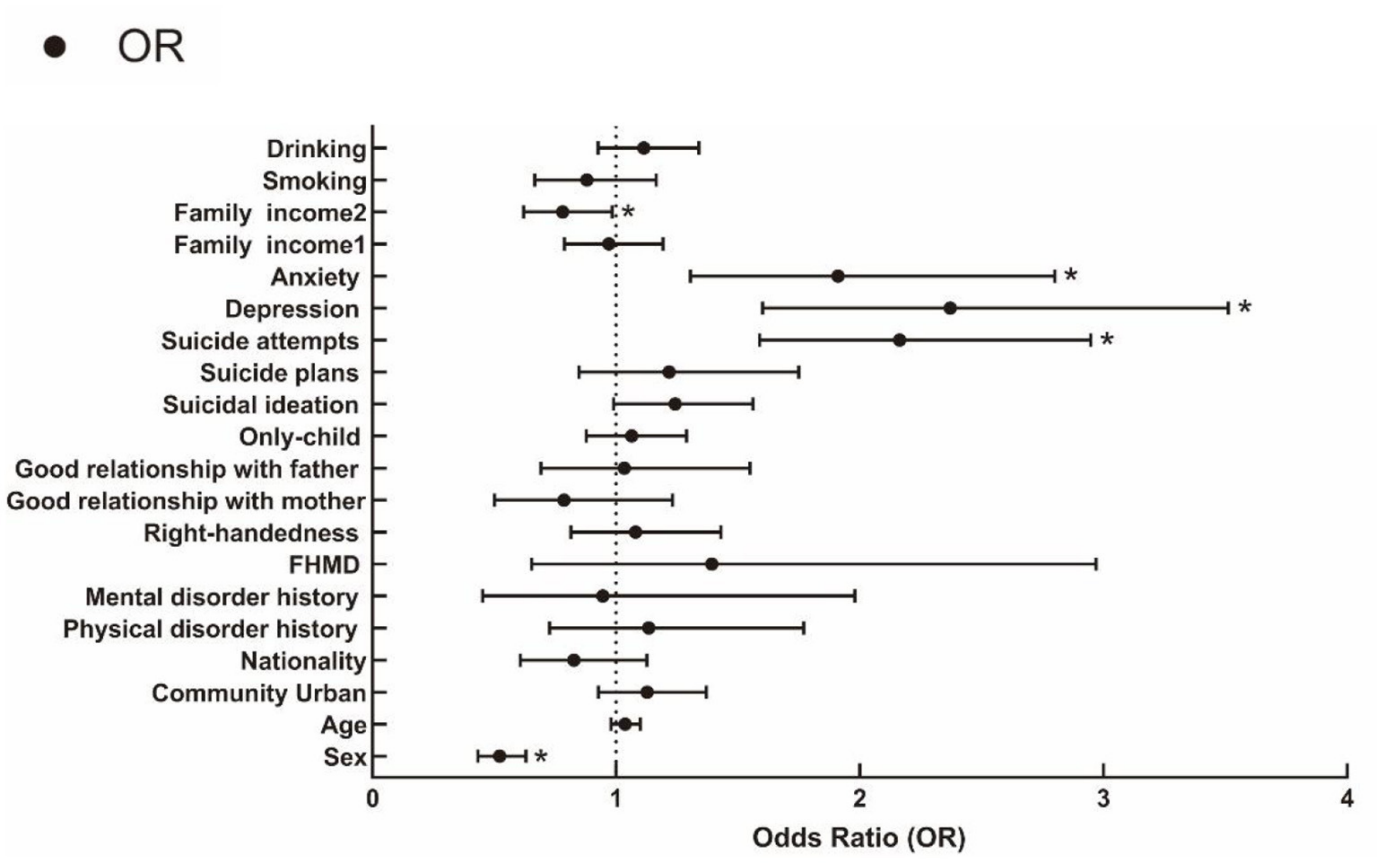
Adjusted odds ratio (OR) of variables associated with cyberbullying using binary logistic regression analysis. OR, odds ratio; FHMD, family history of mental disorders; Family income 1, family income 30,000~70,000 yuan per year; Family income 2, family income over 70,000 yuan per year.

Finally, binary logistic regression analysis showed that sex [Wald = 46.753, df = 1, odds ratio (OR), 0.522; 95% confidence interval (CI) (0.433, 0.629); *p* < *0*.001], suicide attempts [Wald = 23.986, df = 1, OR, 2.164; 95% CI (1.589, 2.948); *p* < *0*.001], depression [Wald = 18.590, df = 1, OR, 2.372; 95% CI (1.602, 3.512); *p* < 0.001] and anxiety [Wald = 11.065, df = 1, OR, 1.911; 95% CI (1.305, 2.800); *p* = 0.001] remained significantly associated with cyberbullying after controlling for confounders. The analysis also showed that a family income over 70,000 yuan per year was a protective factor, compared to a family income under 30,000 [Wald = 4.384, df = 1, OR, 0.781; 95% CI (0.620, 0.984); *p* = 0.036; Table 2; Figure 1].

**Table 2:** Binary logistic regression analysis for variables associated with cyberbullying.

**Variables**	**B**	**S.E**.	**Wald**	**df**	**Sig**.	**Exp (B)**	**95% CI. for EXP (B)**	
							**Lower**	**Upper**
Sex	−0.650	0.095	46.753	1	0.000	0.522	0.433	0.629
Age	0.037	0.030	1.539	1	0.215	1.037	0.979	1.099
Community urban	0.120	0.099	1.454	1	0.228	1.127	0.928	1.370
Nationality	−0.190	0.158	1.454	1	0.228	0.827	0.607	1.126
Physical disorder history	0.126	0.227	0.306	1	0.580	1.134	0.726	1.770
Mental disorder history	−0.056	0.377	0.022	1	0.883	0.946	0.452	1.980
FHMD	0.331	0.387	0.734	1	0.392	1.393	0.653	2.970
Right-handedness	0.077	0.143	0.285	1	0.593	1.080	0.815	1.430
Good relationship with mother	−0.241	0.229	1.109	1	0.292	0.786	0.501	1.231
Good relationship with father	0.034	0.206	0.027	1	0.870	1.034	0.691	1.549
Only-child	0.062	0.098	0.401	1	0.527	1.064	0.878	1.289
Suicidal ideation	0.217	0.117	3.465	1	0.063	1.243	0.989	1.562
Suicide plans	0.197	0.185	1.134	1	0.287	1.218	0.847	1.749
Suicide attempts	0.772	0.158	23.986	1	0.000	2.164	1.589	2.948
Depression	0.864	0.200	18.590	1	0.000	2.372	1.602	3.512
Anxiety	0.648	0.195	11.065	1	0.001	1.911	1.305	2.800
Family income			5.488	2	0.064			
Family income 1	−0.030	0.106	0.083	1	0.773	0.970	0.788	1.193
Family income 2	−0.247	0.118	4.384	1	0.036	0.781	0.620	0.984
Smoking	−0.128	0.143	0.805	1	0.370	0.880	0.665	1.164
Drinking alcohol	0.108	0.094	1.308	1	0.253	1.114	0.926	1.340

FHMD, family history of mental disorders; Family income 1, family income 30,000–70,000 yuan per year; Family income 2, family income over 70,000 yuan per year.

## 4. Discussion

In this study, we investigated participants' cyberbullying experience over the past 12 months. Our study revealed a prevalence of 7.82%, showing that cyberbullying is quite common among Chinese college students. Additionally, cyberbullying was positively related with depression, anxiety, and suicidal behaviors; being female, having a good relationship with parents and having a higher family income showed to be protective factors.

The prevalence of cyberbullying was 7.82% in this study, while previous studies have reported a prevalence ranging from 5.1 to 55.3% in college students (26–29). A study in a sample of 471 US college students showed that 10% had been cyberbullied (26), and another study in the US that recruited 799 participants reported that 8.6% were victims of cyberbullying (27). The different sample size and cultural background may contribute to the discrepancy in the prevalence; for example, in the US, people might be more open to share feelings and experiences, they may be more willing to report cyberbullying victimization, while Chinese people tend to keep things to themselves, some might even feel shameful to share those feelings. One study in a Turkey college reported a high cyberbullying prevalence of 55.3% (29), and this might be due to the fact that the researchers focused on lifetime cyberbullying which might be higher than the prevalence in the past 12 months. On the other hand, data from a Spanish University and a Bolivian University showed a lower cyberbullying prevalence of 5.1% (28). This might be partly due to variations in definitions and personal understanding of cyberbullying, as well as different cultural and economic backgrounds of various regions and nationalities (30). And for Chinese college students, Huang et al. found a high prevalence of cyberbullying up to 64.32% (10), which was apparently much higher than in the present study, it can also be partly explained by the differences in definition of cyberbullying. We emphasized the intentional aggression and the repeated feature of cyberbullying in the present study, while Huang et al. included making fun of others as cyberbullying, for example, in that case, the participants might perceive such behaviors as being aggressive, even though the perpetrator might be unintentional. The difficulty to compare the prevalence of cyberbullying in different studies emphasizes the importance of a unifying definition of cyberbullying. Moreover, the difference in sample size (897 vs. 8,098) might also contribute to the diversity of prevalence of cyberbullying.

Generally, the prevalence of cyberbullying in college students (or young adults) is lower than in adolescents (14.9–36.5%) (17). Researchers have projected that adolescence is a vital period for learning social interaction with others in appropriate ways. However, adolescents' experience of both biological and social changes as well as negative emotions also reach a peak during this special period, including aggression (31, 32), risk taking and impulsivity (33) therefore, they might have more difficulty in social communication, especially chatting with strangers online, which could possibly explain the higher prevalence in adolescents.

Interestingly, we found that being male and low family income were independent risk factors for cyberbullying. Consistent with former studies (10, 34, 35), males were more likely to become cyberbullying victims. It is potentially associated with higher impulsivity of males (10, 36). On the other hand, male students spent more time on online games (37), which lead to higher risk of suffering cyberbullying (38); and males tend to play confrontational, aggressive games online, which made them more likely to suffer from cyberbullying while seeking excitement and satisfaction (39). Family income was found to be positively associated with self-esteem in both teenagers (40) and college students (41), students with low family income were more likely to have low self-esteem, which was associated with cyberbullying (39). Low family income was also associated with insufficient family support (35), which is another risk factor in cyberbullying (42). In addition, compared with female students, male students had lower self-esteem (39), this might also contributed to the different role of sex in cyberbullying.

In the present study, we found that cyberbullying victims were more likely to have depression or anxiety, which is consistent with previous studies (12, 43) and shows that cyberbullying is related to negative mental states. Previous studies have shown that cyberbullying victims are more likely to be anxious, depressed, and have suicidal ideation and attempts (44, 45). MC Martínez-Monteagudo et al. found that up to 72.2% of the victims had high levels of anxiety, and 68.1% reported high levels of depression (12). These association have been observed not only in adults, but also in adolescent samples (46, 47). In addition, compared with traditional forms of bullying, cyberbullying might contribute to more intense negative feelings, like helplessness, fear (48), and social anxiety (49); hence, cyberbullying experience might affect depressive symptoms even more than traditional bullying (47). Moreover, in a study conducted among Swiss and Australian students, researchers found that cyberbullying victims from both countries suffered from depression, and the association was not moderated by country, which suggests that the relation is common in different cultural backgrounds (47).

One study among college students claimed that cyberbullying victimization had negative impact on people's present life and that cyberbullied participants had higher levels of anxiety than the general population (28). Typically, cyberbullying has been found to be associated with social anxiety (45); one longitudinal study proposed that cybervictimization might be a risk factor for social anxiety among adolescents (13). In addition, as has been mentioned, social anxiety might be a partial moderating element in cyberbullying related depression (50). In our study, anxiety was found to be significantly associated with cyberbullying. Other researches have provided similar evidence that cyberbullied individuals present high levels of anxiety, stress (12) and social anxiety (13), and they also show more social avoidance and discomfort in social situations in general (51). Researchers have pointed out that adolescents who lack social skills in real world interactions tend to have more social fears and might choose digital platforms to communicate with others since it is limited to the internet, which makes it more likely to get involved in cyberbullying (52). On the other hand, cyberbullying victimization may lead to even higher levels of anxiety for those who are already suffering from social anxiety (53), making it a vicious circle.

Several previous studies have also focused on the connection between cyberbullying and suicidality (54, 55), and they have reported that cyberbullying victims show more suicidal ideation and attempts than non-victims (54). Other previous studies have shown significant associations between cyberbullying and suicide behaviors (56, 57), such as suicidal ideation (58–63), suicidal plans (57), and suicidal attempts (61, 62, 64). Similarly, our study showed that cyberbullying victims were more likely to have suicide attempts. One previous research showed that cyberbullying had an average causal effect of 4.16% on suicidal attempts (64), and another study in 20, 406 high school students found that victims of cyberbullying were 4 times more likely to develop suicide ideation compared to non-victims (65). While depression has been widely accepted to be directly related to suicidal risks, several studies have shown that depressive symptoms might work as a mediating factor in the association between cyberbullying experience and suicidal behaviors (66, 67). Using mediation analyses, it has also been suggested that psychological distress associated with cybervictimization completely mediates the relationship between cyberbullying and suicidality (54); depressive symptoms significantly mediates the relation between the intensity of being cyberbullied and suicide ideation (67), and state anxiety mediates the relationship between cyber-victimization, cyber-aggressiveness, and depression (11).

As has been shown in previous studies that family support can protect adolescents from being cyberbullied (42), in the current study we also found that good relationship with parents may be a protective factor against cyberbullying. Moreover, family support has been proved to reduce the probability of depressive and anxiety symptoms (68).

There are several limitations we should notice. The first limitation is that this study mainly focused on cyberbullying victims and not cyberbullying aggressors; future studies should address cyberbullying aggressors as well to better understand and prevent cyberbullying. The second limitation is that all the data collected were self-reported information so that recall bias was ineluctable, and although we illustrated the definition of cyberbullying, there may be some deviations due to their own understanding of cyberbullying. Another limitation is that all the data we collected in college students were from the same province; therefore, care should be taken when extending the conclusions into other age groups and provinces. Finally, due to the cross-sectional design, only correlation between cyberbullying and psychological problems can be drawn, we cannot draw any causality conclusions.

## 5. Conclusion

In conclusion, we found that cyberbullying is very common among college students in Hunan Province, China. We also found that cyberbullying may be associated with depression, anxiety, and suicidal behaviors in this population. This study highlights the importance of paying attention to cyberbullying and addressing anxiety, depression, and suicidal behaviors among college students to better improve their mental health and prevent suicide.

## Data availability statement

The raw data supporting the conclusions of this article will be made available by the authors, without undue reservation.

## Ethics statement

The studies involving human participants were reviewed and approved by Ethics Committee of Second Xiangya Hospital of Central South University. The patients/participants provided their written informed consent to participate in this study.

## Author contributions

XL, YS, and XG were responsible for the study design. HX and CH were responsible for recruiting the participants. KZ and XJ were involved in statistical analysis. XJ, KZ, and MT were involved in manuscript preparation and drafting the paper. CH and XG were involved in editing and revising the manuscript. YS, MT, and XL were responsible for the critical revision of the manuscript. All authors have contributed to and have approved the final manuscript, and agree to submit it for consideration for publication in Frontiers in public health.
